# Dual career support among world-class athletes in Sweden: Performance, education, and employment

**DOI:** 10.3389/fpsyg.2022.1093562

**Published:** 2023-01-04

**Authors:** Claes Nyberg, Stefan Wagnsson, Henrik Gustafsson, Owe Stråhlman

**Affiliations:** ^1^Department of Educational Studies, Sports Science, Karlstad University, Karlstad, Sweden; ^2^Department of Sport and Social Sciences, Norwegian School of Sport Sciences, Oslo, Norway; ^3^Department of Sport Science, Linnaeus University, Kalmar, Sweden

**Keywords:** dual career, student-athlete, sport schools, world-class athetes, performance, education, employment

## Abstract

In order to help talented athletes to combine sport and education, different forms of Dual Career Support (DCS) have been developed in many countries. The effectiveness of these support systems have been debated. Most studies have investigated young athletes in the beginning of their careers, less is known about athletes who reached the highest levels. Therefore, the aim of this study was to explore the extent to which former Swedish world-class athletes have attended dual career sport programs at upper secondary school. A retrospective design was used with 274 former world-class athletes who answered a questionnaire. In order to investigate whether there was a relationship between attending a dual career sport school and athletic characteristics, as well as post career educational and employment outcomes, a series of Chi-square tests were conducted. The main results show that more than half of the athletes did not take part in any DCS. These results indicate that DCS in Sweden is not a decisive factor for success in sports as intended. Half of all participants, regardless of whether they studied at DCS, have studied at university, and all participants had a job at the time of data collection. The majority also consider that their financial situation has improved after their sports career.

## 1. Introduction

Several studies indicate that dual career (i.e., combining education with elite sports) pathways make athletes better equipped for life after their sports career. For example, dual career athletes have larger social networks, a more balanced lifestyle and an identity more strongly linked to other domains than sports (Torregrosa et al., 2015; Linnér et al., 2019). Moreover, some scholars have shown that dual career athletes plan their time in a more effective and economical way (Tekavc et al., 2015), get enhanced retirement planning (Aquilina, 2013) and have greater access to the labor market (Tshube and Feltz, 2015). They can therefore end their sports career with less retirement stress and on a more voluntary basis compared to other athletes (Torregrosa et al., 2015). Dual career can also provide other benefits for student athletes. For example, Debois et al. (2015) have shown that athletes with a successful and long career focus not only on their sports career but also on nonathletic components of life, such as education and employment, which serve as support to athletes during periods of decrease in or involuntary breaks from sports performance achievement. This line of research indicates that dual career can carry numerous psychological and psychosocial benefits for elite athletes. However, the balance of combining elite sport and education can be highly challenging (O’Neill et al., 2013; Stambulova and Ryba, 2014; de Subijana et al., 2015; Ryba et al., 2017; Linnér et al., 2019; Quinaud et al., 2022). For example, at upper secondary sport schools, many elite athletes experience difficulties in balancing their education, sports, and private life (Stambulova et al., 2015; Ryba et al., 2017). It seems common that dual career athletes most often choose the educational options that are the easiest to combine with elite sports (Küttel et al., 2020) and have to sacrificing educational success when integrate elite sport with education (Cosh and Tully, 2014). Moreover, the high demands placed on the athlete when trying to combine sports and education can cause stressful study situations, which in turn may result in psychological stress, overtraining and injuries (Gustafsson et al., 2008; O’Neill et al., 2013; Sisjord and Sorensen, 2018; Sorkkila et al., 2020). Harrison et al. (2022) and Linnér et al. (2021) have also shown that the higher the level of education, the more challenging it becomes for student athletes to combine studies with elite preparatory training, and that student-athletes need coping strategies and support to get optimal dual career balance.

Research has clearly shown that talented adolescent athletes may face many difficulties when trying to combine an elite sports career with a corresponding educational career. In order to address these difficulties and prepare athletes for a post-sport career, different forms of dual career support programs (e.g., elite sport school and study program support) for talented adolescent athletes have been developed in various countries (Lund, 2014; de Subijana et al., 2015; Stambulova et al., 2015; Tshube and Feltz, 2015; De Bosscher et al., 2016). As a result, a great deal of economic resources have been spent on dual career support programs across Europe in an attempt to help talented youth athletes attain the highest level in both their sports and education endeavors (European Commission, 2012), highlighting the need to examine the usefulness of dual career support programs.

In Sweden there are two types of dual career support programs: (1) Dual career support-international level (DCS-IL), and dual career support-national level (DCS-NL). Students who are admitted to DCS-IL are selected based on an assessment that they can reach the international elite sport level, and those who are admitted to DCS-NL are selected based on the assessment that they can at least reach the national elite sport level (Ferry, 2014). These designated sports school is an important part of the talent development program (Stambulova et al., 2015).

Attending a dual career sport program may seem advantageous, but researchers have shown some shortcomings, calling into question the usefulness of such programs. Sisjord and Sorensen (2018) have shown that athletes’ expectations to perform at a high level decreased over a three-year period when attending a dual career support program (an upper secondary skiing school in Norway). The majority of participants reported that they intended to quit their sports career immediately after graduation. In addition, only 8% of Dutch students who took part in a dual career sport program at upper secondary school reached an international senior top level in their sports (van Rens et al., 2015). This shows the challenges in dual career sports program and talent development environments.

Although the intention of a dual career sport program is to help talented athletes achieve the highest possible level in sports as well as in education, the two aims can be difficult to combine. Several studies (van Rens et al., 2015; Küttel et al., 2020; Sæther et al., 2022) have found that most student athletes who stated that they wanted to reach the international top level prioritized their sport over their studies when studying at a sport school with a dual career sport program profile, contradicting the intention of such a program. A specific example is found in a study by Ronkainen et al. (2018) who showed that ice hockey coaches affected student athletes to prioritized their athletic career over their studies. Athletes who focused exclusively on their athletic career have been shown to be at risk of athletic identity foreclosure, which can lead to adaptation difficulties when ending one’s sports career (Grove et al., 1997). Contradicting the main goal of dual support programs, van Rens et al. (2015) found that talented athletes who had not taken part in a dual career sport program at upper secondary school achieved better results in both sports and studies, and were more likely to continue studying at university. Moreover, they were significantly more motivated to do well in school compared to those who had attended a dual career sport program.

Despite some findings showing that dual careers may be advantageous for athletes (Debois et al., 2015; Torregrosa et al., 2015), the main findings above support the notion that dual career sport programs are struggling to facilitate help talented student athletes in reaching their full potential in sports as well as in education. Storm et al. (2021) believes that it is important that upper secondary sports school provide coordinated approaches to ensure both sport and study development for student-athlete. Therefore, it is important to develop the support for the whole environment in which the student-athlete are embedded, and not only focus on the individual student-athletes.

However, research in this area has almost exclusively focused on talented student athletes at the beginning of their sports career (Stambulova et al., 2015; van Rens et al., 2015; Sisjord and Sorensen, 2018; Davis et al., 2019; Sorkkila et al., 2020), while specific studies including world-class athletes are lacking. For example, the study by van Rens et al. (2015) of 242 talented athletes included only athletes at the beginning of their sports career, with a mean age of 21. The response rate was 20%, which is considered too small to be able to draw any general conclusions, and as most talented athletes will likely not become elite athletes, this study is limited in its inclusion of only successful elite athletes at the beginning of their sports career. This makes it difficult to draw safe conclusions about the importance of dual career sport programs for successful athletes, as the goal of these programs is to help talented athletes attain the highest level in sports while also succeeding in their education endeavors (European Commission, 2012). Hence, it is especially important to investigate whether or not those who have reached the absolute top senor elite level have taken part in a dual career sport program at upper secondary school, and if so, to examine whether or not it has been beneficial.

One exception is a study by Emrich et al. (2009), looking at 199 successful elite athletes, was conducted included 2004 Summer Olympics and 2006 Winter Olympics participants. The purpose was to investigate whether there were any differences between school performance, post-school occupational prospects, and competitive success between those who had taken part in DCS and those who had not. The results show no differences between the groups in school performance. For the participants in the 2004 Summer Olympics there were no differences between DCS participants and non-participants in competitive performance, but for the participants in the 2006 Winter Olympics there was a connection between competitive performance success and being a DCS participant. These results are interesting but limited, as only 16% of the study group had taken part in DCS consistently and the response rate was only 32.6%. From this point of view, the aim of the current study is to explore the extent to which former Swedish world-class athletes have attended a dual career sport program at upper secondary school. The following research questions was put forward: Are there significant association between school options (DCS-IL, DCS-NL, and MSS) and gender, type of sport, sports performance, post-education, opportunities for higher education, employment attainment and post-athletic economic situation?

## 2. Materials and methods

### 2.1. Participants

A sample of all 431 former Swedish world-class athletes (42% women and 58% men) who had participated in the Summer or Winter Olympic Games, the World Cup, the European Championships, or corresponding championships (e.g., the European Cup, Grand Slam tournaments) between 2002 and 2012 were asked to participate in the study. The inclusion criteria entailed athletes who had competed on an international top elite level between 2002 and 2012 and had ended their elite athlete career during the time of the data collection. The inclusion criteria was also merits at least Nordic champion medalist or placement 4–10 in European championship, European Championship medalists, World Cup medalist, Olympic medalists, World Champion medalists, World record holder and placing 1–3 in major competitions (i.e., Wimbledon in Tennis, Masters in golf).

Initially, a total of 274 participants (58% men and 42% women, mean age = 39.92, SD = 6.25) responded to the questionnaire (64% response rate). The participants were divided into 41 sports, both summer and winter, of which 27 were individual sports, athletics (*n* = 16), swimming (*n* = 13), biathlon (*n* = 11), snowboarding (*n* = 11), cycling (*n* = 10), cross-country skiing (*n* = 9), wrestling (*n* = 8), sport shooting (*n* = 7), golf (*n* = 6), ski cross (*n* = 4), table tennis (*n* = 4), tennis (*n* = 4), archery (*n* = 4), fencing (*n* = 1), gymnastics (*n* = 2), walking (*n* = 9), alpine skiing (*n* = 8), figure skating (*n* = 4), horse riding (*n* = 4), freestyle alpine (*n* = 3), ice skating/short track (*n* = 3), modern pentathlon (*n* = 2), taekwondo (*n* = 2), trialthon (*n* = 2), boxing (*n* = 1), judo (*n* = 1), weightlifting (*n* = 1), and 14 were team sports, football male (*n* = 19), football female (*n* = 18), ishockey female (*n* = 17), ice bandy (*n* = 13), curling (*n* = 12), handball female (*n* = 10), ishockey male (*n* = 9), sailing (*n* = 7), canoe (*n* = 6), handball male (*n* = 6), bob (*n* = 2), rodel (*n* = 2), rowing (*n* = 2), volleyball (*n* = 1). All but three participants were born in Sweden. Nearly half (48%) of the study group had grown up in a sparsely populated or small town, a third (31%) in a medium-sized to larger city, and a fifth (21%) in a metropolitan area. Half of the participants (51%) had university as their highest level of education, and 37% had upper secondary school and 4% had compulsory school as their highest level of education. The majority (61%) were employed at the time of data collection, and almost a quarter (35%) had their own company, while a minority (4%) were off duty, studying or were not employed for unknown reasons. Due to internal missing data, different numbers of participants will be presented in the results (e.g., 11 participants had not answered the question regarding what type of upper secondary school they studied at. Consequently, only answers from 263 participants are reported). We have based our division (in individual and team sports) on the same reason as Lupo et al. (2015), who described how team sports have a competition schedules spread over several month, while individual sport are more focused to specific periods; this can have an impact on how the student is able to manage the combination of studies and elite sports.

### 2.2. Instrument

In the current study we asked the following questions. Weather respondent attended a DCS-program or not was measured by the question, “*Have you studied at any DCS-program?*” Sport performance was measured by the question, “*What is your highest sporting achievement during your elite sports career?”* with free text as an answer option. Highest level of education was measured by the question, “*What is your highest completed education?*.” If elite sports career has been a hinder for higher education was measured by the question, “*Elite sports career hindered your opportunities for higher education?*.” Employment after elite sport career was measured by the question, “*What is your current occupation?*” Economic situation was measured by the question, “*Have your financial circumstances changed after your elite sports career ended?*” Those respondents who answered that their economic situation had changed were asked to fill out at which form the economic situation had changed with the answer options, “*For the better*” and “*For the worse*.”

### 2.3. Procedure

After ethical approval from the Regional Ethics Board (Dnr 2016/428), a questionnaire was sent out to former world-class athletes. The respondents’ addresses were found in various public address registers online, while some were obtained from different sports associations *via* telephone and e-mail. Participants were sent a letter informing them of the purpose of the project, how to complete the questionnaire, and ethical issues such as voluntary participation, anonymity, and the right to withdraw at any time if desired without consequences. Respondents were asked to return the completed questionnaire, in an enclosed postage-paid envelope, within 10 days. After 3 weeks from the first contact, the research team sent a first reminder letter. The second and final reminder was sent 3 weeks after the first one.

### 2.4. Data analyses

Data analyses were conducted using the statistics program SPSS 26.0. A series of Chi-square tests were conducted in order to investigate whether there is a relationship between school options (i.e., DCS-IL, DCS-NL, and MSS) and gender, type of sport, level of sporting success, and post-career outcomes (i.e., highest level of education, current employment, and economic situation). The significance level for the analysis was set to 0.05.

## 3. Results

### 3.1. Participant characteristics

The participants, representing former successful Swedish world-class athletes at a senior international level, were divided into three levels based on their highest success in their sport (Table 1). These categories are similar to the athlete classification system referred to in Swann et al. (2015) based on four types of elite performer: “world-class elite,” “successful elite,” “competitive elite,” and “semi-elite.” The most successful athletes in this study belong to Level 1, “world-class elite,” the second-most successful ones to Level 2, “successful elite,” and the least successful ones to Level 3, “competitive elite.” The category “semi-elite” incorporates athletes below the top elite standard level, and was therefore not included in this study. As seen in Table 1 more than half of the participants were world-class athletes, all of whom have won a medal at one of the major international championships (i.e., Olympics or World Cup/Championships) or have broken a world record.

**Table 1:** Participants athlete classification based on their highest sporting success

1. World-class elite athletes (*n* = 140; 51%)	2. Successful elite athletes (*n* = 87; 32%)	3. Competitive elite athletes (*n* = 47; 17%)
Olympic medalists, World Champion medalists, World record holder, placing 1–3 in major competitions (i.e. e., Wimbledon, Masters in Golf).	European Championship medalists, World Cup medal, placement 4–10 Olympics or World Cup.	Placement 4–10 European Championship, Nordic Championship medalist. Ranking 10–12 Olympics or World Cup, ranking 4–10 World Cup, National Championship medal, champion abroad, ranking 20 or less in the Olympics.

### 3.2. Attendance of dual career support program

Regarding what kind of upper secondary school the participants had attended, results showed that 44% (*n* = 116) had attended some kind of dual career support program in upper secondary school (i.e., DCS-IL or DCS-NL), while the largest proportion (56%, *n* = 147) had attended a mainstream upper secondary school (MSS). Notably, even though all participants had reached an international elite level, only 31% (*n* = 82) of them had attended schools that were supposed to attract/select the best athletes (e.g., DCS-IL), indicating that DCS-IL is not a decisive factor in Swedish athletes reaching the highest international level in their sport (Table 2).

**Table 2:** The relationship between attending a dual career sport program/school and athletic characteristics as well as post career educational and employment outcomes.

Athletic characteristics and post career outcomes	DCS-IL		DCS-NL		MSS		Total	*Χ*^2^/value of *p*	Cramer’s *V*
	*n*	(%)	*n*	(%)	*n*	(%)			
**Gender**								1.81/0.405	0.083
Men	52	(34)	18	(12)	81	(54)	151 (100)		
Women	30	(27)	16	(14)	66	(59)	112 (100)		
**Type of sport**								13.69/0.001	0.228
Team	18	(18)	18	(18)	63	(64)	99 (100)		
Individual	64	(39)	16	(10)	84	(51)	164 (100)		
**Sporting success**								1.43/0.839	0.052
World class elite	42	(31)	20	(15)	72	(54)	134 (100)		
Successful elite	27	(32)	8	(10)	48	(58)	83 (100)		
Competitive elite	13	(29)	6	(13)	26	(58)	45 (100)		
**Highest level of education**								1.89/0.389	0.090
University	36	(30)	21	(14)	79	(56)	136 (100)		
Upper secondary school	33	(35)	12	(13)	50	(53)	95 (100)		
**Limited opportunities for higher education**								9.21/0.056	0.134
Yes	17	(40)	4	(9)	22	(51)	43 (100)		
Partly	33	(33)	18	(18)	48	(49)	99 (100)		
No	29	(25)	11	(10)	76	(65)	116 (100)		
**Employment**								4.84/0.304	0.097
Employed	45	(29)	19	(12)	91	(59)	155 (100)		
Own company	30	(34)	11	(12)	48	(54)	89 (100)		
Off duty/student	5	(46)	3	(27)	3	(27)	11 (100)		
**Economic situation**								1.03/0.596	0.075
Better	41	(30)	18	(13)	77	(57)	136 (100)		
Worse	19	(38)	6	(12)	25	(50)	50 (100)		
**Total**	82	(31)	34	(13)	147	(56)	263 (100)		

DCS-IL = dual career support international level; DCS-NL = dual career support national level; MSS = mainstream secondary. Due to internal missing data, different numbers of participants may vary within each category. Cramer’s V: 0.10—weak association, 0.30—moderate, 0.50—strong association.

#### 3.2.1. Gender and type of sport

As far as gender differences are concerned, the results showed that the proportion of boys and girls was largely evenly distributed across the different school forms (Table 2). However, a medium strong significant relationship (Pallant, 2020) was noted between the type of sport and attending a dual career sport program. Results in Table 2 show that more than double the proportion of individual athletes have studied in a dual career support program at an international level compared to team sport athletes. Concerning the other two school forms (i.e., DCS-NL and MSS) the distribution was more even distributed, with slightly more team sports in DCS-NL and more individual sports in MSS (Table 2). Thus, the results indicate that it is more common for individual athletes than team sport athletes in Sweden to choose to attend DCS-IL.

#### 3.2.2. Sporting success

As shown in Table 2, 46% of the world-class elite athlete group attended some form of dual career support program (i.e., DCS-IL or DCS-NL), while the majority studied at a MSS. Almost the same distribution was seen for the other levels of sporting success. Hence, there was no significant relationship between sporting success and attending any dual career sport program, indicating that attending neither DCS-IL nor DCS-NL is crucial for Swedish athletes in order to reach the top elite level in sports.

#### 3.2.3. Education

Results in Table 2 show a non-significant relationship between attending a dual career support program and advancement to higher education. Although the results show that the proportion who advance to higher education is higher among athletes who have studied in DSC-IL compared to DSC-NL, almost the same distribution was detected for those who reported having upper secondary school as their highest level of education, indicating that DSC-IL is no better than DSC-NL in creating good conditions for advancing to higher education. Moreover, findings show a marginally significant association between attending a dual career support program and perceived limited opportunities for higher education. A deeper analysis of the adjusted residuals indicates that it is mainly those who studied on DCS-IL programs who experience these limitations. In summary, these results indicate that attending a dual career support program is not crucial for Swedish athletes in order to advance to a higher level of education, or to avoid difficulties with educational attainment.

#### 3.2.4. Employment

Results in Table 2 show a non-significant relationship between attending a dual career support program and employment. Notably, the proportion of employed or having one’s own company was slightly higher among those who had attended a MSS than those attending any dual career sport program. To sum up, these results indicate that attending a dual career support program is not crucial for Swedish athletes in order to be employed or start their own company.

#### 3.2.5. Economic situation

As seen in Table 2, the majority of the former elite athletes believed that their financial situation had improved after their elite sports career while about a quarter considered that their financial situation had become worse after ending their elite sports career. These patterns appeared regardless of whether they had attended DCS-IL, DCS-NL, or MSS, indicating that attending a dual career support program is not crucial for Swedish athletes’ economic situation post-athletic career.

## 4. Discussion

The main aim of this study was to explore the extent to which former Swedish world-class athletes had attended a dual career sports program at upper secondary school, and whether this support was related to their past sports performance, post-education and employment attainment, and post-athletic economic situation. Although the study group was represented by former Swedish world-class athletes, the main findings showed that only 44% had taken part in some form of DCS. Moreover, although all participants had reached the international level in their respective sport, results revealed that only 31% of them had attended schools (i.e., DCS-IL) supporting dual careers for athletes in order for them to reach the international top level.

In order to help talented adolescent student athletes attain the highest level in both their sport and their education, different forms of DCS have been developed in many countries (Lund, 2014; de Subijana et al., 2015; Stambulova et al., 2015; Tshube and Feltz, 2015; De Bosscher et al., 2016). Existing research has illustrated that DCS struggles in helping these athletes reach their full potential in sport and education in parallel (van Rens et al., 2015; Ronkainen et al., 2018; Sisjord and Sorensen, 2018), which may call into question its usefulness. However, most of the research conducted in this area has focused on young athletes at the beginning of their sports career (O’Neill et al., 2013; Stambulova et al., 2015; van Rens et al., 2015; Sisjord and Sorensen, 2018; Davis et al., 2019; Sorkkila et al., 2020), and, there is a lack of studies on athletes who have reached the world-class at senior level. In order to fil this gap of knowledge, we investigated Swedish former world-class athletes who had reached at least an international level, according to the classification system devised by Swann et al. (2015). Based on the proposed classification half of the participants belong to Category 1, “world-class elite athletes.” A third belong to Category 2, “successful elite athletes,” and almost a fifth to Category 3, “competitive elite athletes.” We can therefore state that this study include “the best of the best athletes” at a senior level in Sweden.

The results show no difference between those who had taken part in any DCS and those attending mainstream upper secondary school (MSS). These results are consistent with the findings by van Rens et al. (2015) and Emrich et al. (2009) that talented athletes attending elite sport schools that offer DCS are not more likely to achieve the highest sporting level compared to those attending MSSs. These results are surprising; while DCS-IL in Sweden has the goal of preparing student athletes to do well in international championships (Stambulova et al., 2015), our results indicate that it is not crucial to study within DCS-IL in order to reach an international elite level. The fact that as many as over half of our research group did not take part in any DCS means that one can question the importance of DCS for our research group’s success in sports.

Emrich et al. (2009) suggest that upper secondary DCS foremost selects early-maturing athletes and thereby leaves out those who are late-maturing, as mentioned in the literature as the Relative Age Effect (RAE; Wattie et al., 2008). Research shows that the RAE is an important factor for an athlete to be selected for elite sports teams (Helsen et al., 2005; Hancock, 2017) For example Sæther et al. (2017) found that RAE was a decisive factor to attending a sport specialization program at lower and upper secondary school in both Norway and Sweden. Thus, the RAE (Cobley et al., 2009) may explain why only 44% of our study group had taken part in DCS. Van Rens et al. (2015) found that student athletes who had taken part in any DCS at upper secondary school practiced their sport for more hours per week at a younger age, and had a higher sport performance level at the start of upper secondary school, than did their counterparts at MSSs. This can create early sporting specialization thinking in order to enter upper secondary school with DCS, and can result in a risk of later sporting stagnation and dropout from sports among young athletes (Emrich et al., 2009). Modest sports results at a young age due to late biological maturity may be one of the reasons why over half of our survey group did not take part in any DCS.

We believe that a danger of the RAE in terms of athletic success in early years is that young, promising athletes who develop late in sports may lose interest and quit when they discover they have not been selected for upper secondary school with DCS. It can also lead to increased training at a young age to accelerate one’s sports development (Atkinson and Goodway, 2021), which goes against several studies that show support for the risks of early specialization, demonstrating that these young athletes are more likely to suffer various overuse injuries, burn out, eating disorders, later sports stagnation, and early dropout from sports (DiSanti and Erickson, 2019; Rugg et al., 2021; Mosher et al., 2022). Empirical studies also support one of the most heralded notions, that athletes do not need to specialize early in order to reach elite status as adults (Côté et al., 2009; DiSanti and Erickson, 2019; Atkinson and Goodway, 2021). This can be compared to our survey group, in which the majority started specializing upon entering upper secondary school between 15 and 16 years of age, which may be considered a reasonable age to specialize. So the question is how many prospective elite athletes quit prematurely, and how many are forced into early specialization in order to be selected for DCS at upper secondary school? More than half of the athletes in the current study have not taken part in DCS at upper secondary school, which may indicate that taking part in DCS is not crucial in order to achieve success in sports and may indicate that these athletes has not been forced to early specialization, or it was their and their parents’ decision, after considering, perhaps, the existing costs and benefits—further studies are needed to find an appropriate response to this situation. Atkinson and Goodway (2021) consider that many parents believe that their children have to specializing early to become an elite athlete and to get better sporting conditions, but the current findings might indicate that this is not the case.

The intention of DCS is to help talented youth athletes attain the highest level in both their sports and their education (European Commission, 2012). Recent research in Denmark (Storm and Eske, 2022) indicate that athletes at DCS did not have lower grades than non-athletes students. Our results show that almost 60% of the athletes (i.e., DCS-IL, DCS-NL, and MSS) have university as their highest education level, with slightly more for those attending DCS-NL and MSSs. Among those who attended DCS-IL, it is more common to only have upper secondary school as one’s highest education level. The reason for this may be that the requirements for sports performance are higher for students at DCS-IL, thus forcing them to invest in sport at the expense of studies at upper secondary school. Several studies (van Rens et al., 2015; Sæther et al., 2017; Ronkainen et al., 2018; Küttel et al., 2020) also show that student athletes who take part in any DCS tend to prioritize their sport over their studies. Consequently, the DCS athletes, attain lower educational results in upper secondary school, and are less motivated to study at university compared to their counterparts at MSSs. One reason for this may be that the focus on sports performance is greater when one is involved in DCS (Cosh and Tully, 2014; Küttel et al., 2020). Our results also show that more of those who have taken part in DCS-IL and DCS-NL agree that their sports career hindered their opportunities for higher education, which may also be because the DCS athletes needed to prioritize their sport over studies. This may lead to some shortcomings for student athletes, as athletes who focus exclusively on their athletic career have been shown to be at risk of athletic identity foreclosure and adaptation difficulties when ending their sports career (Grove et al., 1997; Park et al., 2013). It may be the case that those in our study who did not participate in DCS at upper secondary school were allowed to have a calmer sports development and as a result were able to focus more on their studies in parallel, which may have benefited their elite sports retirement process. On a positive note, it can be stated that more than half of all participants, regardless of whether they studied at DCS, have studied at university, and all participants had a job at the time of data collection. The majority also consider that their financial situation has improved after their sports career, which may also indicate that everyone, regardless of whether they took part in DCS, has succeeded in making a stable income after their elite sports career.

The main results of this study show that more than half of the survey group, comprised of former Swedish world-class athletes, did not take part in any DCS at upper secondary school, even though the goal of DCS is to help talented athletes attain the highest level in both their sports and education endeavors (European Commission, 2012). These results may indicate that DCS in Sweden is not a decisive factor for success in sports as intended. Our results also show that half of our survey group had university as their highest education level, and that there is no difference between those who had attended DCS and those who had not. These results may also indicate that DCS in Sweden does not result in study success any more than among those student athletes who attainted MSS. In their study, van Rens et al. (2015) also found that attending DCS at upper secondary school did not influence the highest attained sport performance levels of talented athletes. They also found that DCS students attained lower educational levels in both secondary school and further education. Our results indicate that talented athletes at the beginning of their sports careers do not necessarily need to attend DCS during upper secondary school to succeed in reaching an international elite level in their sport and simultaneously succeed in their studies. These results may additionally indicate that MSSs have also developed supporting facilities for over half of our study group. More research is needed in order to draw such a conclusion. Based on governmental funding, an annual of 4 million euros (43 million SEK) is spent on DCS in Sweden. In addition, various types of subsidies from the student home municipalities (Regeringskansliet, 2020) and regional and local investments are added to the DCS system. When such a great deal of economic resources are spent on dual career support programs across Europe, and in Sweden, to try to help talented adolescent athletes attain the highest level in both their sports and education endeavors (European Commission, 2012), these results should be surprising. There is a need to review the recruitment of young athletes to DCS at upper secondary school. After all more successful elite athletes should be involved in DCS if extensive financial resources are invested in this support system.

### 4.1. Limitations and future research

Our study covers all Swedish world-class athletes who won medals at international events between 2002 and 2012, and we traced back what type of upper secondary school they attended. As our survey group answered a questionnaire, we did not have the opportunity to ask follow-up questions. This makes it difficult to determine why the respondents did or did not take part in DCS and we lack the respondents’ experiences and opinions about DCS that are important to find out. In a future study it would be desirable to interview world-class athletes about their views on DCS at upper secondary school and to determine how world-class athletes have handled the combination of studies and elite sports without DCS both at upper secondary school and at university, as a university path is another important factor in preparing student athletes for post-sports careers.

## 5. Conclusion

Most research on dual career support have focused on young athletes in the beginning of their sport careers. Little is known about successful athletes who reached the highest elite levels. In this study, including 274 former world-class athletes who had reached an international level, we found that only 44% had taken part in some form of DCS. Although all participants had reached the international level in their respective sport, results revealed that only 31% of them had attended specialized schools (i.e., DCS-IL) supporting dual careers for athletes in order for them to reach the international top level. Moreover, the results indicate that it is more individual athletes than team athletes who had studied at DCS-IL, and it is mainly those who studied on DCS-IL programs who perceived limited opportunities for higher education. Our results also show that more than half of each group (i.e., DCS-IL, DCS-NL, and MSS) have university as their highest education level, with slightly more for those attending DCS-NL and MSSs. Among those who attended DCS-IL, it is more common to have only upper secondary school as one’s highest education level. The reason for this may be that the requirements for sports performance are higher for students at DCS-IL, thus forcing them to invest in sport at the expense of studies at upper secondary school. It was also found that more than half of all participants, regardless of whether they studied at DCS, have studied at university, and all participants had a job at the time of data collection. The majority also consider that their financial situation has improved after their sports career, which may also indicate that everyone, regardless of whether they took part in DCS, has succeeded in making a stable income after their elite sports career. Our results show that taking part in DCS is not crucial for young talent student athlete in order to achieve success in sports and education.

## Data availability statement

The raw data supporting the conclusions of this article will be made available by the authors, without undue reservation.

## Ethics statement

The studies involving human participants were reviewed and approved by The Regional Ethics Board, Uppsala. The patients/participants provided their written informed consent to participate in this study.

## Author contributions

CN, SW, HG, and OS contributed to conception and design of the study. CN organized the database and wrote the first draft of the manuscript. CN and SW performed the statistical analysis. SW, HG, and OS, wrote sections of the manuscript. All authors contributed to the article and approved the submitted version.

## Conflict of interest

The authors declare that the research was conducted in the absence of any commercial of financial relationships that could be construed as a potential conflict of interest.

## Publisher’s note

All claims expressed in this article are solely those of the authors and do not necessarily represent those of their affiliated organizations, or those of the publisher, the editors and the reviewers. Any product that may be evaluated in this article, or claim that may be made by its manufacturer, is not guaranteed or endorsed by the publisher.
